# *Shape2SAS*: a web application to simulate small-angle scattering data and pair distance distributions from user-defined shapes

**DOI:** 10.1107/S1600576723005848

**Published:** 2023-07-28

**Authors:** Andreas Haahr Larsen, Emre Brookes, Martin Cramer Pedersen, Jacob Judas Kain Kirkensgaard

**Affiliations:** a Department of Neuroscience, University of Copenhagen, Copenhagen, Denmark; b University of Montana, Missoula, Montana, USA; c Niels Bohr Institute, University of Copenhagen, Copenhagen, Denmark; d Department of Food Science, University of Copenhagen, Copenhagen, Denmark; Argonne National Laboratory, USA

**Keywords:** small-angle scattering, *Shape2SAS*, geometrical shapes, teaching, simulation, virtual experiments, pair distance distributions, structure factors, form factors, multi-contrast particles

## Abstract

*Shape2SAS* is a web application that allows researchers and students to build intuition about and understanding of small-angle scattering. It is available at https://somo.chem.utk.edu/shape2sas. The user defines a model of arbitrary shape by combining geometrical subunits, and *Shape2SAS* then calculates and displays the scattering intensity and the pair distance distribution, as well as a visualization of the user-defined shape. Simulated data with realistic noise are also generated. Here, it is demonstrated how *Shape2SAS* can calculate and display the different scattering patterns for various geometrical shapes, such as spheres and cylinders. It is also shown how the effect of structure factors can be visualized. Finally, it is indicated how multi-contrast particles can readily be generated, and how the calculated scattering may be used to validate and visualize analytical models generated in analysis software for fitting small-angle scattering data.

## Abstract 2

*Shape2SAS* allows the user to simulate solution small-angle scattering data and pair distance distributions from user-defined shapes. The program is available as a web application, and is useful for teaching and tutorials, as well as for checking the validity of analytical form factors.

## 1. Introduction

We introduce *Shape2SAS*, a website for simulating small-angle scattering (SAS) data and pair distance distributions from various shapes. *Shape2SAS* is readily available as a web application (https://somo.chem.utk.edu/shape2sas) implemented using the *GenApp* framework (Savelyev & Brookes, 2019 ▸) for constructing graphical user interfaces (GUIs) for scientific software. The user can construct particles of arbitrary shape by combining geometrical subunits, *e.g.* two spheres and a cylinder to form a dumbbell. The user can thus compare calculated scattering and pair distance distribution from different particle shapes, such as cylinders and spheres, or more elaborate particles shapes, such as dumbbells or spheres coated with smaller spheres. The dimensions and excess scattering-length density of the particle (*i.e.* contrast between particle and average solvent scattering-length density) can be adjusted to build intuition about how these affect the resulting scattering. The user can also investigate the effects of polydispersity or structure factors.

The website is particularly useful for educators when organizing introductory or advanced courses in SAS data analysis. Due to its nature as a stand-alone web application, the only requirement is a web browser. The program can likewise be used for testing analytical form factors, as demonstrated in this article.

*Shape2SAS* applies a Monte Carlo based method previously implemented in the program *McSim* (Hansen, 1990 ▸, 2014 ▸) (see the comparison between *Shape2SAS* and *McSim* in Table S1 of the supporting information).

*Shape2SAS* also outputs simulated data using an empirical error model. The simulated data provide an idea about how exposure time, concentration, contrast and particle size affect the noise in data. Furthermore, the simulated data can be used in tutorials on how to analyze SAS data, *e.g.* using programs for fitting analytical form factors, such as *SasView* (https://www.sasview.org/), *SASfit* (Breßler *et al.*, 2015 ▸) or *WillItFit* (Pedersen *et al.*, 2013 ▸), or programs for *ab initio* modeling (Franke & Svergun, 2009 ▸; Grant, 2018 ▸).

## 2. Applied small-angle scattering theory

In this section, we provide the theoretical and computational basis for *Shape2SAS*, and account for the implementation of this.

### 2.1. Normalized scattering from identical particles in solution

The normalized SAS from a diluted sample of randomly oriented identical particles can be calculated as a double sum over all scatterers in each particle (Debye, 1915 ▸):

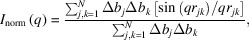

where Δ*b*_*j*_ is the excess scattering length of the *j*th scatterer, *r*_*jk*_ is the distance between the *j*th and *k*th scatterers, and *q* is the momentum transfer [ 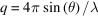 , where 2θ is the scattering angle and λ is the wavelength of the incoming X-rays or the de Broglie wavelength of the incoming neutrons]. *N* is the number of scatterers in each particle. We assume point scatterers, so the effect of atomic form factors is neglected. The user provides excess scattering-length densities as input, ΔSLD_*i*_ = Δ*b*_*i*_/*V*, where *V* is the effective volume of one scatterer. The effective volume is given as the particle volume (*V*_p_) divided by the number of scatterers: *V* = *V*_p_/*N*. The point density is kept constant in all subunits, so *V* is also constant. This normalized scattering is the form factor, *P*(*q*), of the particle.

### 2.2. The pair distance distribution

By binning the scattering pairs after their pair distances, *r*_*jk*_, the double sum can be reduced to a single sum over the number of bins:

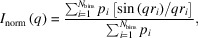

where 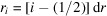 and d*r* is the bin width. The term *p*_*i*_ is the number of pairs in each bin, weighted by the product of their excess scattering lengths:

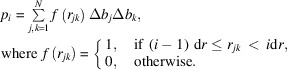

This is also a double sum but sin(*qr*)/*qr* is not evaluated for each distance.

The pair distance distribution *p*(*r*) is the continuous limit of 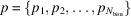 , as *N*_bins_ → ∞ and *N* → ∞. In this limit, the contributions from the self terms (*j* = *k*) are negligible. Therefore, the self terms are excluded from the sums in *Shape2SAS*, and *p*(0) = 0 is added.

The largest distance in the particle, *D*_max_, is given as output, along with the radius of gyration (Glatter, 1977 ▸; Guinier & Fournet, 1955 ▸):

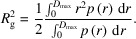

The output *p*(*r*) is normalized, so the maximum is unity.

### 2.3. Polydispersity

Polydispersity implies a distribution of sizes of the particles (Pauw *et al.*, 2013 ▸). *Shape2SAS* allows for inclusion of a simple Gaussian size distribution. The size distribution is implemented by introducing a size-scaling parameter, *s*, which scales all distances in the user-defined shape (*r* → *sr*). The parameter *s* is normally distributed, with a mean of unity (μ_*s*_ = 1) and standard deviation (σ_*s*_) set by the user.

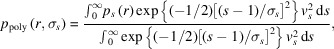

where *p*_*s*_(*r*) is the pair distance distribution after scaling all distances with *s* and *v*_*s*_ = *s* ^3^ is a relative (unitless) volume, which is included to account for the fact that the scattering from a particle is proportional to the square of the molecular volume. The relative volume is an approximation and is only exact for spheres. The integrals are evaluated numerically with upper and lower limits of 1 ± 3σ_*s*_

While this is a simplification of the many ways in which a sample can exhibit polydispersity, the implementation is simple and efficient, and allows users and students to explore the consequences of polydispersity in SAS data.

### 2.4. Structure factors

Interparticle interactions can be expressed in the form of structure factors, and the normalized scattering intensity can be expressed as a product of the form factor and the structure factor (Breßler *et al.*, 2015 ▸):

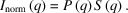

So far, two structure factors are implemented in *Shape2SAS*: a hard-sphere structure factor (Kinning & Thomas, 1984 ▸), describing interparticle repulsion, and a 2D fractal structure factor, describing particle aggregation (Larsen *et al.*, 2020 ▸). The decoupling approximation is also applied to account for non-spherical or polydisperse particles (Kotlarchyk & Chen, 1983 ▸; Larsen *et al.*, 2020 ▸). Although such interparticle interactions would affect the effective pair distance distribution, the program only applies the structure factor on the calculated scattering intensity. That is, if a structure factor is opted for, the output *p*(*r*) is from non-interacting particles, whereas *I*(*q*) is from a sample of interacting particles. The *p*(*r*) of the interacting particles can *e.g.* be generated in *BayesApp* (Hansen, 2012 ▸), which is likewise available as a web application in *GenApp*.

### 2.5. Interface roughness

A particle can be modeled by combining geometrical subunits. If the subunits have different contrasts, there may be discrepancies between the sharp boundaries between subunits of the model and the softer and more fluent boundaries between components in an actual particle. The interface roughness can be modeled by including a surface roughness factor (Als-Nielsen & McMorrow, 2011 ▸), which effectively smears the interfaces:

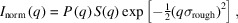

where σ_rough_ is a smearing parameter. In *Shape2SAS*, surface roughness only affects *I*_norm_(*q*), not *p*(*r*). Such terms have been applied to fit scattering from liquid–vapor interfaces (Als-Nielsen, 1986 ▸), microemulsions (Skar-Gislinge & Arleth, 2011 ▸; Foster *et al.*, 2008 ▸) and protein–lipid nanodiscs (Skar-Gislinge & Arleth, 2011 ▸; Barclay *et al.*, 2022 ▸).

### 2.6. Simulation of data with an empirical noise model

Besides the pair distance distribution and the normalized scattering, *Shape2SAS* also outputs simulated data, *I*_sim_(*q*). First, the forward scattering is calculated, so that it scales with particle volume, *V*_p_, and volume fraction (concentration of the sample), η: 

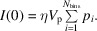

The last factor depends on the contrast, and if all subunits have the same contrast then 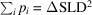 . The scaled scattering is given as *I*(*q*) = *I*(0)*I*_norm_(*q*).

The errors, σ_sim_(*q*), are estimated using an empirical model based on small-angle X-ray scattering (SAXS) data (Sedlak *et al.*, 2017 ▸), using the scaled scattering, *I*(*q*), and the relative exposure time as input (Appendix *A* [App appa]). When using the default parameters, the errors resemble those from a typical synchrotron SAXS experiment. To simulate laboratory-source data, the exposure time should be reduced, *e.g.* by an order of magnitude (Sedlak *et al.*, 2017 ▸). Future efforts are needed to simulate realistic small-angle neutron scattering (SANS) errors (see *Discussion* [Sec sec5]). The simulated data, *I*_sim_(*q*), are sampled stochastically from normal distributions with means *I*(*q*) and standard deviations σ_sim_(*q*).

## 3. Implementation

### 3.1. Architecture

**Figure 1:**
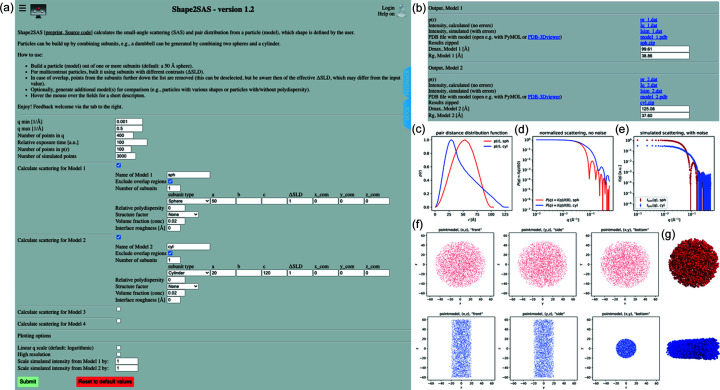
Simulating scattering from a sphere (red) and a cylinder (blue) using *Shape2SAS*. (*a*) GUI input parameters. (*b*) GUI output parameters. (*c*) Pair distance distributions. (*d*) Calculated normalized scattering. (*e*) Simulated scattering intensities with noise. (*f*) 2D projections of the models. (*g*) 3D models rendered in *PyMOL* (http://www.pymol.org).

The program is designed for ease of use and ease of maintenance. Input parameters are provided via an online GUI (Fig. 1 ▸). Inputs are read by a Python wrapper script. The wrapper calls Python functions that perform the calculations and return output files, plots and values to the wrapper. Finally, the wrapper returns the output to the GUI, which displays them for the user.

### 3.2. Description of core functions

A core element of *Shape2SAS* is the Python function that generates points from a given subunit. First, the limits of the geometric subunit are defined in either Cartesian, polar or spherical coordinates. Then, random uniformly distributed points are generated in the volume defined by the subunit. Lastly, if selected, the points are shifted to a new center of mass. The volumes of the subunits are calculated, and the number of points inserted in each subunit is adjusted, so that the point density is constant (see *Applied small-angle scattering theory* [Sec sec2]). The total number of points in the model (composed of one or more subunits) is *N* = 3000 by default, which provides a rapid result. For higher precision, *N* can be increased. The computational time goes as *N* ^2^. For *N* = 3000 and two models, the results and plots are available within 6 s, whereas *e.g.* for *N* = 9000 and two models, the results and plots are available within 14 s. The SAS intensity calculated from the same particle will vary stochastically, with larger differences at higher values of *q*. By increasing *N*, the differences are decreased; depending on the *q* range of interest, *N* should be adjusted to balance computational time against precision. With 5000 points, the calculated intensity for a 50 Å sphere is precisely reproduced up to approximately *q* = 0.2 Å^−1^ (Fig. S1 of the supporting information). In the case of overlap between subunits, the points from the subunits furthest down the list of subunits are removed, *e.g.* if the user chooses a 30 Å sphere with ΔSLD = −1 as the first subunit and a 50 Å sphere with ΔSLD = 1 as the second subunit, the resulting particle will be a core–shell particle with a 30 Å core with ΔSLD = −1 and a shell with thickness of 20 Å and ΔSLD = 1 (examples in Fig. S2). In the overlap region, only the points from the first subunit remain. Exclusion of points from overlapping subunits can be exploited in multi-contrast situations (see *Example 3* [Sec sec4.3]).

Another core element is the calculation of all distances in a model. This step is time and memory consuming, and one of the computational bottlenecks of the program. However, using the *NumPy* (Harris *et al.*, 2020 ▸) function meshgrid(), all distances between 3000 points are calculated in less than a second on the *GenApp* server. Another computational bottleneck is the calculation of *p*(*r*). This is carried out using the *NumPy* function histogram(). When polydispersity is included, several histograms are calculated, and in that case *Shape2SAS* uses the histogram1d() function (https://github.com/astrofrog/fast-histogram), which is faster than *NumPy*’s histogram(). A polydisperse *p*(*r*) is thus calculated in a few seconds.

### 3.3. User interface

A key goal of the project is accessibility and ease of use. Therefore, the program is implemented as a web application, meaning that no installation is required. This is optimal for use in courses and tutorials. The program is part of the *GenApp* (Savelyev & Brookes, 2019 ▸) vision for making SAS software available for everyone.

The user builds up a model of a predefined set of geometric subunits, currently including sphere, triaxial ellipsoid, cylinder, disc, cube, cuboid, hollow sphere, hollow cube, cylindrical ring and discoidal ring. The geometrical parameters of the subunits can then be changed from the default values, along with their contrast and center of mass. A model consists of the collected points in the volume spanned by the subunits. By default, points are deleted from overlapping regions, but this is optional as mentioned above. If a structure factor is selected, the scattering contribution from the structure factor will be displayed along with the total scattering.

The user can choose to calculate *p*(*r*) and *I*(*q*) from additional models (up to four), and the procedure is the same as for the first model. The *p*(*r*) and *I*(*q*) from the models are plotted together in the GUI, and can thus be directly compared without having to plot the data in third-party software. The simulated data can be scaled in the plot for easy visual inspection.

All output data [*p*(*r*), *I*_norm_(*q*) and *I*_sim_(*q*)], as well as plots and a 3D model for visualization (in PDB format), can be downloaded from the web interface for further analysis, plotting *etc*.

### 3.4. Documentation and validation

All menus and input boxes in the GUI are described with help text, which is shown by hovering the mouse over the elements. The source code is documented with extensive comments in the code, including documentation of all functions. Central references are provided directly in the GUI. Each model is visualized as a 3D point model using a *Jmol* (http://www.jmol.org/) plugin and as 2D projections. *Shape2SAS* models and structure factors have been tested against analytical models using *SasView* (https://www.sasview.org).

Source code is available on GitHub (https://github.com/ehb54/GenApp-Shape2SAS) under the GNU General Public License v3.0.

## 4. Examples of use

These examples are designed to be relevant in the context of research as well as research-based teaching. The first example showcases how *Shape2SAS* can be used for generating intuition about SAS from different shapes, and the second example demonstrates the effect of inter-particle interactions. The last example demonstrates how more complex particles can be built and how *Shape2SAS* can be utilized to test analytical form factors.

### 4.1. Example 1: comparing scattering from particles of different shape

*Shape2SAS* can be used to quickly calculate and compare *p*(*r*) and *I*(*q*) from particles with various shapes. One example is the scattering from a sphere with a radius of 50 Å, and a cylinder with a radius of 20 Å and a length of 400 Å (Fig. 1 ▸). Such an example could help a student build intuition about the scattering and pair distance distribution functions for *e.g.* spherical, elongated or hollow bodies. Moreover, *R*_g_ and *D*_max_ are calculated and displayed in the GUI for quick comparison.

### 4.2. Example 2: hard-sphere structure factor

**Figure 2:**
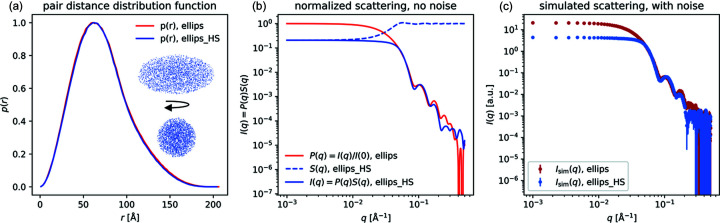
Scattering intensity from ellipsoids with and without interparticle repulsion. (*a*) Pair distance distributions from two instances of a prolate ellipsoid of revolution, without interparticle repulsion. Inset: 2D projections of the ellipsoid along the minor and major axes. (*b*) Normalized scattering intensity from the ellipsoids with and without interparticle repulsion as described by a hard-sphere structure factor. (*c*) Simulated scattering, with noise.

*Shape2SAS* can add inter-particle interactions to the scattering using built-in structure factors. One example is shown in Fig. 2 ▸, where the scattering from a sample of ellipsoids of revolution (minor axis 50 Å and major axis 100 Å) was calculated with and without interparticle interaction, described by the hard-sphere structure factor and the decoupling approximation, with a hard-sphere radius of 70 Å and a volume fraction of 0.2. Such an exercise could help students or researchers to understand the effect of structure factors and recognize interparticle interactions in measured SAS data.

### 4.3. Example 3: validating analytical form factor

**Figure 3:**
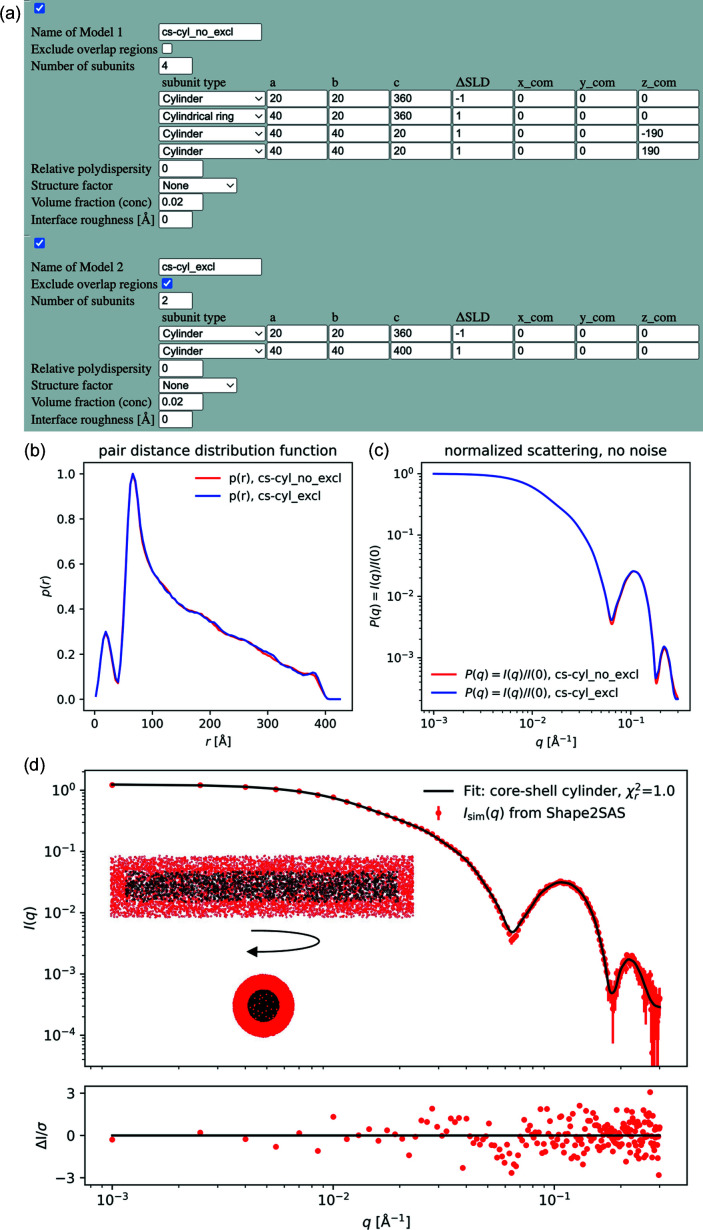
Core–shell cylinder data, simulated in *Shape2SAS* and fitted with an analytical model. (*a*) Core–shell cylinder input, without use of exclusion and with use of exclusion. (*b*) Pair distance distribution from *Shape2SAS*. (*c*) Normalized SAS intensity. (*d*) Simulated data fitted with the analytical model core_shell_cylinder from *SasView*. Inset: 2D projections of the core–shell cylinder.

*Shape2SAS* can be used when developing analytical form factors. By generating the same shape in *Shape2SAS* as that of the analytical model, the simulated data from *Shape2SAS* can be fitted. In this example, a core–shell cylinder was simulated (core radius 20 Å, core length 360 Å, shell thickness 20 Å, core contrast −1 and shell contrast +1). The example also showcases how multi-contrast particles can be generated in two ways in *Shape2SAS*. In the first approach, the core–shell cylinder is generated by combining non-overlapping subunits: a cylinder core, a hollow cylindrical shell and two small cylinders with shifted center of mass as end caps [Fig. 3 ▸(*a*)]. In the second approach, a large shell cylinder and a smaller core cylinder are combined, and points from the shell cylinder are removed from the overlapping region [Fig. 3 ▸(*a*)]. Both result in the same model, *p*(*r*) and *I*(*q*) [Figs. 3 ▸(*b*) and 3 ▸(*c*)]. If there is overlap, and exclusion of overlapping points is not opted for, then the contrast in the overlap region will effectively be the sum of contrasts of the overlapping subunits.

The simulated data were fitted with the analytical model core_shell_cylinder from *SasView* (https://www.sasview.org/sasmodels/model/core_shell_cylinder.html). The data were well described by the model [Fig. 3 ▸(*d*)] and the model parameters were refined to values consistent with the input parameters (core radius 20.10 ± 0.07 Å, core length 363 ± 2 Å, shell thickness 20.1 ± 0.1 Å and shell contrast +0.97 ± 0.01). The errors are standard deviations. The core contrast was fixed at −1, since the combination of fitting core contrast and shell contrasts, and then scaling, gave high correlation between the parameters.

The demonstrated procedure can be valuable for testing new and more complex analytical models (Jakubauskas *et al.*, 2019 ▸), and for visualizing them as bead models.

This workflow is helpful when coding analytical models for SAS from particles in solution. While analytical models are often ideal for implementation in a model-refinement framework (such as *SasView*), *Shape2SAS* offers a simple, graphical and intuitive manner for testing the implementation and its accuracy – in real space.

## 5. Discussion

*Shape2SAS* is useful for demonstrations and tutorials, which has been showcased by three examples. *Example 1* [Sec sec4.1] compared the SAS from a sphere with the SAS from a cylinder; *Example 2* [Sec sec4.2] demonstrated the effect of interparticle repulsion via a hard-sphere structure factor; and *Example 3* [Sec sec4.3] showcased how more advanced shapes can be built, with various excess scattering-length densities, and how *Shape2SAS* can be used to test analytical form factors.

When carrying out analysis of the simulated data, discrepancies between input parameters and refined parameters may occur from the stochastic nature of *Shape2SAS* and the limited number of simulated points [Fig. 3 ▸(*d*)]. Moreover, if polydispersity is fitted, this is probably implemented differently in analytical models and may result in discrepancies.

*Shape2SAS* does not distinguish between SAXS and SANS. To make the distinction, the scattering-length densities should be provided in real units, reflecting either electron density or neutron scattering length density. Moreover, the empirical error model was derived from SAXS data. In SANS, the incoherent scattering should be considered along with resolution effects (Pedersen *et al.*, 1990 ▸). Furthermore, SANS data are often collected in several settings and subsequently merged (Pedersen, 2002*a* ▸), which should be reflected in the error model.

Other programs exist that can calculate the theoretical scattering from various shapes using form factors, including *SASfit* (Kohlbrecher & Breßler, 2022 ▸), *Irena* (Ilavsky & Jemian, 2009 ▸) and *SasView* (https://www.sasview.org/). These programs can also be used to build intuition about the scattering from particles of various shapes, and the effect of changing model parameters or adding structure factors. *Shape2SAS* is complementary to these programs: (i) by being available as a web application; (ii) by performing fast comparison of scattering and pair distance distributions from particles of various shapes; (iii) by automatically visualizing the particles; (iv) by generating simulated data with an empirical error model, which can be used as a virtual experiment for tutorials and to build intuition about some of the sources of noise in a SAS experiment; and (v) by being able to calculate scattering from particles that cannot be described by an analytical form factor.

*Shape2SAS* is one among many programs for making virtual experiments (Lefmann *et al.*, 2008 ▸), some of which can also generate 2D X-ray (Bergbäck Knudsen *et al.*, 2012 ▸; Franke *et al.*, 2020 ▸) or neutron (Willendrup & Lefmann, 2021 ▸) scattering data. *Shape2SAS* focuses on the data-analysis step, after reduction from 2D to 1D data, and after buffer subtraction. Common for such virtual-experiment software is the goal of preparing the user for best use of valuable beam time, and for helping the user to better understand and analyze the measured data. *Shape2SAS* excels in accessibility, meaning that virtual data can be generated in seconds, with no installation requirements.

In principle, the *Shape2SAS* input parameters could be transformed into variable parameters, and by addition of a scaling and a constant background, such a modified program could be used for fitting. It has previously been shown that Monte Carlo bead modeling approaches are useful in SAS analysis (Pedersen, 2002*b* ▸), especially for generating complicated models that are difficult to describe through analytical form factors [*e.g.* perforated vesicles (Pedersen *et al.*, 2012 ▸) or protein–lipid complexes (Pedersen *et al.*, 2022 ▸)]. However, other programs applying the same principles have been developed with fitting in mind, including *CDEF* (Deumer *et al.*, 2022 ▸) and *SPONGE* (https://github.com/bamresearch/sponge), and we recommend use of these for fitting. *CDEF* applies the same principle of binning scattering pairs as *Shape2SAS*, whereas *SPONGE* applies the Debye formula directly, making it more accurate at the cost of longer computational times. When fitting actual data, we also encourage parametrizing the model to reflect physical properties rather than geometrical, to be able to better constrain and validate the refined parameters, *e.g.* with biophysical assays [see *e.g.* Skar-Gislinge & Arleth (2011 ▸) and Larsen *et al.* (2018 ▸)]. This is not possible in *Shape2SAS* and would typically have to be adjusted from case to case, which is easier without a GUI. *Shape2SAS* may, however, be downloaded, and functions reused by developers, to accommodate specific needs.

In summary, *Shape2SAS* makes SAS theory intuitive, visual, playful and accessible for both new and experienced users.

## Supplementary Material

Supporting information. DOI: 10.1107/S1600576723005848/jl5064sup1.pdf

## Appendix A Empirical error model

The errors were generated following the previously outlined protocol (Sedlak *et al.*, 2017 ▸):

(1) Compute a theoretical SAS profile, *I*(*q*), as described in the main text.

(2) Scale *I*(*q*). Scaling is done such that the scattering from a 50 Å sphere with ΔSLD of unity (default parameters) has a forward scattering of approximately unity.

(3) Multiply the scaled *I*(*q*) by the relative exposure time (GUI input) to get *I*_*s*_(*q*).

(4) Calculate the variance using *c* = 0.85 and  , which define the constant scattering intensity of the buffer, *I*_buf_(*q*) = *cI*_*s*_(*q*_arb_) (Sedlak *et al.*, 2017 ▸):

In the original error model (Sedlak *et al.*, 2017 ▸), the number of pixels per *q* bin, *N*(*q*), was approximated by *kq*. This is, however, only valid up to a certain *q* value, *q*_*t*_, which depends on detector size and geometry. Therefore, we use a modified empirical expression for *N*(*q*) that better describes the number of pixels per *q* bin at higher values of *q*:

Values of *k* = 4500,  , *a* = 3 and *b* = 0.6 Å^−1^ were used in *Shape2SAS* to approximate a synchrotron SAXS setup with centered beam center on a PILATUS 2M detector (Fig. S3).
