# Supplementary material for: Shape2SAS: a web application to simulate small-angle scattering data and pair distance distributions from user-defined shapes
Source: J Appl Crystallogr. 2023 Jul 28;56(Pt 4):1287–94. doi: 10.1107/S1600576723005848 (PMC10405587; doi:10.1107/S1600576723005848)
Supplement: Supplementary file 1 [file j-56-01287-sup1.pdf]

JOURNAL OF
APPLIED
CRYSTALLOGRAPHY

Volume 56 (2023)

Supporting information for article:

Shape2SAS: a web application to simulate small-angle scattering data and pair distance distributions from user-defined shapes

Andreas Haahr Larsen, Emre Brookes, Martin Cramer Pedersen and Jacob Judas Kain Kirkensgaard

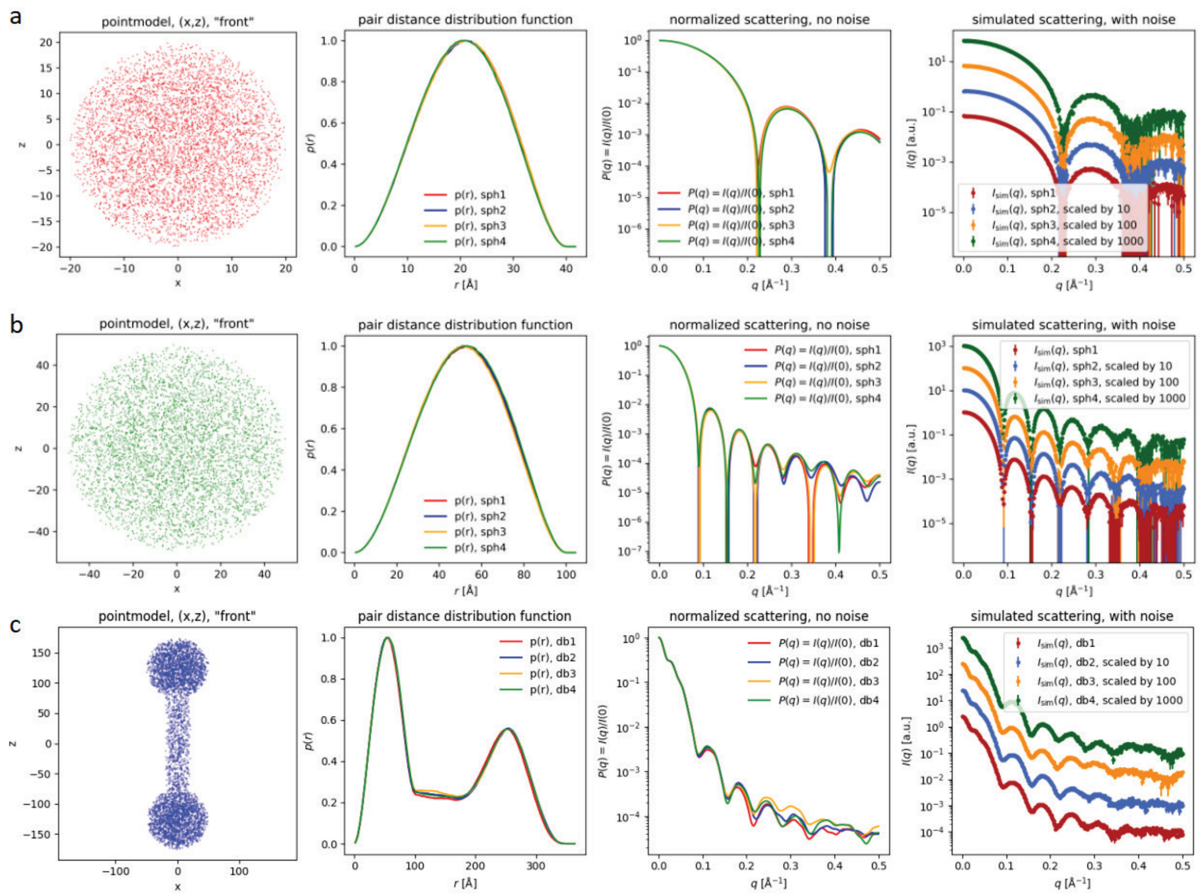

Figure S1. Precision of Shape2SAS when using 5000 points. Calculated pair distance distribution and intensity for five repeated simulations. (a) Sphere with radius 20 Å. (b) Sphere with radius 50 Å. (c) Dumbbell composed of a cylinder with radius 20 Å and length 200 Å, and two spheres of radius 50 Å, with their center of mass shifted ±125 Å.

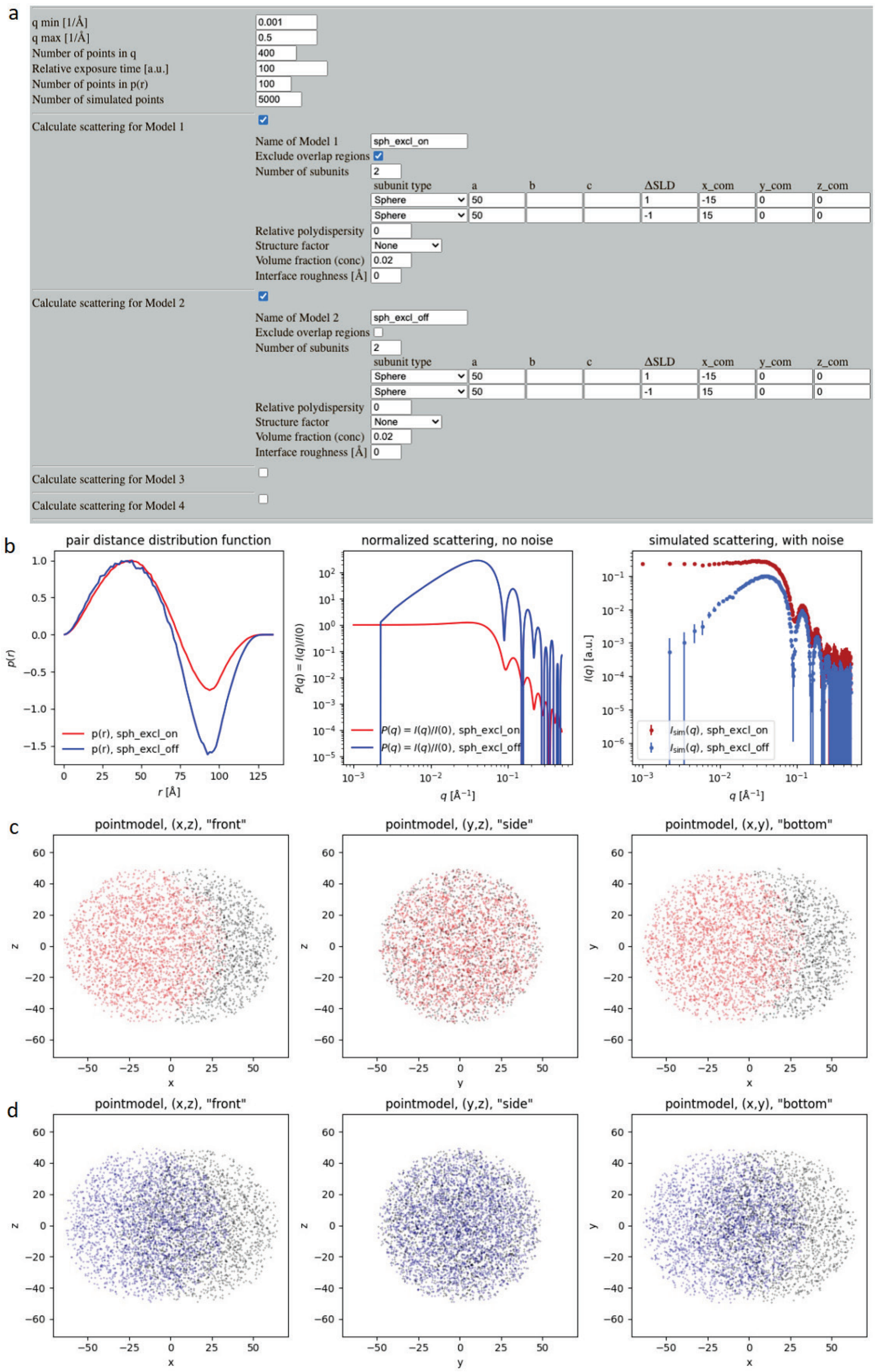

Figure S2. Illustration of 2 models with exclusion of regions enables (red) or disables (blue). (a) Input. (b) Output pair distance distribution and scattering. (c-d) 2D projections of the particles. Red/blue for positive contrast, black for negative.

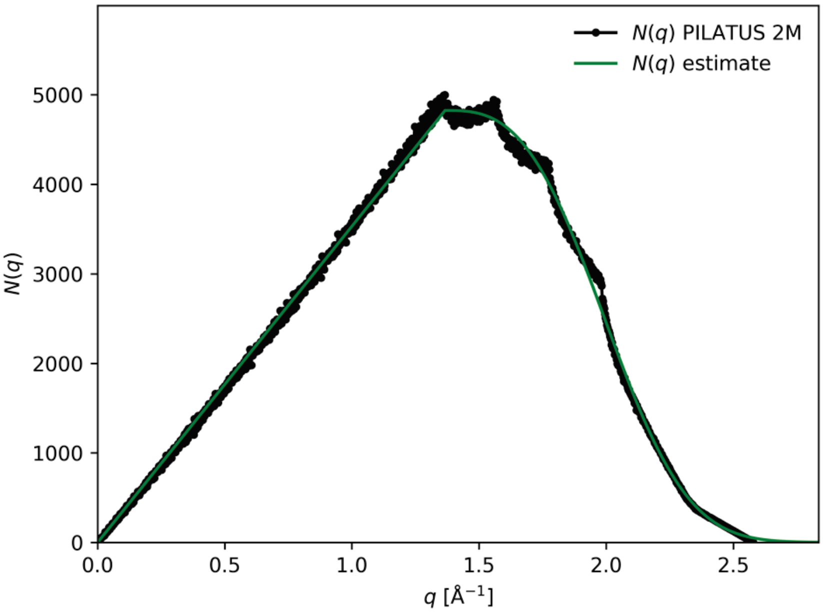

Figure S3. Numerical approximation (black) of the number of pixels per q bin, obtained by simulating a SAXS setup with a PILATUS 2M detector (centered beam, pixel size 172x172 um, 1475x1679 pixels, $\lambda = 1.0$ Å, sample-detector distance 0.5 m, 1000 bins in the $N(q)$ histogram). Analytical approximation (green) plotted on top.

	McSim	Shape2SAS version 1.2
Language	Fortran77+Gnuplot	Python3
Installation	compile from source code	no installation required
GUI	none*	web-application
Max number of models	1	4
Max number of subunits	10	15
Visualization of model(s)	2D projections	2D projections, 3D visualization
Documentation	source code	source code, current paper, web app.
---------- *Additional options* ----------		
Polydispersity	yes (Gaussian)	yes (Gaussian)
Interface roughness	no	yes
Structure factors	none	hard-sphere, aggregation (2D fractal)
---------- *Simulated data* ----------		
Error model	$\sigma(q) = aI(q) + b$	empirical model (Appendix A)
Forward scattering	$I(0) = 1$	depends on contrast, volume and conc.

Table S1: comparison of McSim and Shape2SAS. *There used to be a web interface for McSim [1], but this is no longer available.

References for supplemental information

1. Hansen, S. Update for BayesApp : A Web Site for Analysis of Small-Angle Scattering Data. *J. Appl. Crystallogr.* **2014**, *47*, 1469–1471, doi:10.1107/S1600576714013156.
